# Supplementary material for: Optimization of Z-shaped assembled mortise and tenon joint box culvert connection and structural characteristics
Source: PLoS One. 2025 Jun 5;20(6):e0323950. doi: 10.1371/journal.pone.0323950 (PMC12140261; doi:10.1371/journal.pone.0323950)
Supplement: Attachment — Submitted filename: Reviewer Comments.docx [file pone.0323950.s001.docx]

Reviewer Comments.

This research paper proposes a new type of z-shaped prefabricated tongue-and-groove jointed box culvert, which is analysed using finite element simulation software to provide valuable insights into the practical engineering design of similar combined box culverts. It has certain academic value and practical significance. The research method is reasonable, the data analysis is more rigorous, and the conclusions are basically reliable. However, some important issues should also be addressed before the manuscript is accepted:

General comments:

(1) Whether the parameters of Fig. 1abc can be presented all in 1a.

(2) The introduction of the research background is relatively clear, but in order to elaborate the research significance of ‘how to improve the stability of longitudinal connection of prefabricated box culvert through the use of specialised structural forms and connection methods’, the preliminaries are too much, so it is suggested to modify and adjust.

(3) The references of the current scholars are too old, it is difficult to explain the significance of this research and the unique advantages compared with the existing research, it is recommended to increase a certain amount of newer references.

(4) How the numerical analysis model grid is divided. Explain the difference between Fig. 2 and Fig. 3a?

(5) The use of Ansys Workbench to build the model for finite element numerical simulation is reasonable, but the model simplification assumptions and the impact on the accuracy of the results are not fully explained in the paper. The assumptions made in the model building process need to be elaborated.

(6) Detailed data are provided in the table of material parameters of the model, but the basis for selecting some parameters (e.g. modulus of elasticity of reinforcement, Poisson's ratio, etc.) is not mentioned. The source of the parameters should be explained, whether they are based on actual tests, normative standards or previous research experience, in order to enhance the credibility of the study.

(7) The conclusion part summarises the main findings and results of the study, but the presentation is rather general, and it is suggested that some quantitative results should be added to elaborate the innovativeness of the article.

(8) There are some grammatical and expressive inaccuracies, such as‘the maximum deformation and stress concentration in the side wall are prominently observed at the ends and armpits of the joint, specifically at the connection between the two culvert joints. The phrase ‘are prominently observed’ in ‘are prominently observed’ is rather rigid, and can be replaced by ‘are mainly located’, which is more natural. It is recommended to proofread the whole paper carefully to optimise the language expression and improve the readability of the paper in order to eliminate some language errors and typos.

In summary, the paper has some value in terms of research content and methodology, but it needs minor revision for the above problems. It is hoped that the authors can take the review comments seriously and revise and improve the paper meticulously. If the revised paper can meet the above requirements, I think it is expected to be published in this journal.
